# Prior Diagnosis of COVID Has No Increased Complications in Total Joint Arthroplasty

**DOI:** 10.7759/cureus.27974

**Published:** 2022-08-13

**Authors:** Brandon E Lung, Taha M Taka, Megan Donnelly, Maddison McLellan, Kylie Callan, Leo Issagholian, Wilson Lai, David So, William McMaster, Steven Yang

**Affiliations:** 1 Orthopedic Surgery, University of California, Irvine, Irvine, USA; 2 Orthopedic Surgery, University of California, Riverside, Riverside, USA; 3 Orthopedic Surgery, University of California, Irvine School of Medicine, Irvine, USA

**Keywords:** covid-19 pandemic, postoperative complications, average length of stay, post-operative joint infection, covid 19, arthroplasty, joint replacement, orthopedic surgery

## Abstract

Introduction

Although a substantial portion of the United States population has been infected with and recovered from Coronavirus Disease-19 (COVID-19), many patients may have persistent symptoms and complications from disease-driven respiratory disease, arrhythmias, and venous thromboembolism (VTE). With institutions resuming elective total joint arthroplasties (TJA), it is unclear whether a prior resolved diagnosis of COVID has any implications on postoperative outcomes.

Methods

All elective TJA performed in 2021 at our institution were retrospectively reviewed and a history of prior COVID+ result recorded. Baseline demographics, days from prior COVID+ result to surgery date, preoperative methicillin-resistant Staphylococcus aureus (MRSA) nares colonization, and laboratory markers were obtained to determine baseline characteristics. Postoperative estimated blood loss (EBL), length of stay (LOS), rate of revision surgery, and discharge destination were compared between groups. Perioperative and postoperative rates of VTE, urinary tract infection (UTI), pneumonia, postoperative oxygen supplementation, cardiac arrhythmia, renal disease, sepsis, and periprosthetic joint infections within six months of surgery were recorded.

Results

Of the 155 elective TJA performed in 2021, 24 patients had a prior COVID+ diagnosis with a mean of 253 days from positive result to surgery date. There were no significant differences in baseline demographics, comorbidities, and preoperative lab markers between groups. Surgeries on patients with a prior COVID+ had a significantly higher EBL (260 vs 175cc), but postoperative outcomes of VTE, UTI, pneumonia, oxygen supplementation requirement, nares MRSA+, cardiac disease, and infection rates between groups were similar. Bivariate logistic regression revealed increased days from COVID+ diagnosis (>6 months) to surgery date were associated with a shorter LOS.

Conclusion

Although a prior COVID+ diagnosis had increased intraoperative blood loss, there were no significant differences in respiratory, infectious, cardiac, and thromboembolic complications up to six months after elective TJA. This study suggests that asymptomatic C+ patients receiving elective TJA do not require more aggressive prophylactic anticoagulation or antibiotic regimens to prevent VTE or perioperative infections. As institutions around the nation resume pre-COVID rates of arthroplasty surgeries, a prior diagnosis of COVID appears to have no effects on postoperative complications.

## Introduction

Due to the COVID-19 nonessential procedure restriction, there was a large decrease in orthopedic procedures during the pandemic. One study estimated that approximately 30,000 primary and 3000 revision hip and knee arthroplasty procedures were canceled each week throughout the COVID-19 nonessential procedure restrictions [1]. As cases resumed, multiple studies have explored the short-term effects of COVID-19 on the perioperative morbidity and mortality of various orthopedic surgeries. A 2020 study by Kayani et al. demonstrated an increased length of hospital stay, more critical care admissions, higher risk of perioperative complications, and increased mortality in COVID-19-positive (C+) patients undergoing hip fracture surgery compared to COVID-19-negative (C-) patients [2]. These results were consistent with other orthopedic surgical outcomes of femur neck and ankle fracture surgeries [3,4].

Several notable complications that were tightly bound to the COVID-19 virus were the increased risk of venous thromboembolism, atrial fibrillation, as well as respiratory issues inherent to the virus. As recently assessed by Forlenza et al., the risk of deep vein thrombosis (DVT) and pulmonary embolism (PE) was significantly higher in COVID-19 patients undergoing total joint arthroplasty (TJA), owing to the hypercoagulability associated with the inflammatory state [5]. Additionally, the study determined a temporal relationship between COVID-19 diagnosis and TJA, with an increased risk of DVT and PE in patients who were diagnosed with COVID-19 one month prior to their operation versus two or three months. This temporalizing trend was also witnessed when assessing the post-operative risk for pneumonia between C+ and C- patients [6,7]. Likewise, a study exploring the complication rates in C+ patients after hip fracture repair demonstrated an increased risk of post-operative atrial fibrillation compared to C- patients [7].

Notably, a rare yet significant complication of joint replacement is the risk of infection of the prosthesis which is a common cause of joint replacement revision. While minimal data has been presented regarding the risk of prosthetic joint infection, previous studies have demonstrated no significant risk of infection in TJA [6,8]. Additionally, the imposed restriction during the COVID-19 pandemic significantly hindered the ability of patients to seek adequate and continuous rehabilitation post-operatively which led to overall worse patient-reported outcomes [9]. Lastly, while the length of hospital stay (LOS) for surgical orthopedic patients has decreased in hopes of limiting the risk of COVID-19 infection, there was a significant increase in LOS in a previous study in C+ hip fracture patients compared to C- patients secondary to increased risk for complications as well as slower rehabilitation and dependence on oxygen supplementation.

While the short-term effects of a recent COVID-19 diagnosis on post-operative outcome have been explored, the effects of a previous C+ diagnosis and recovery on the outcomes of an orthopedic procedure and, more specifically, total joint arthroplasties, remains unclear. As the COVID-19 virus becomes increasingly more ubiquitous, it is important to gain an understanding regarding the complications or lack thereof that previously infected patients may face in future TJA procedures. For this reason, the objective of this study is to establish the complication risks of previous COVID-19-positivity on the postoperative outcomes of total joint arthroplasty.

This study aims to identify any significant differences in prosthetic joint infections, DVT and PE incidence, post-operative oxygen requirement, estimated blood loss (EBL), and LOS between prior asymptomatic C+ and C- patients undergoing TJA.

## Materials and methods

All elective TJA performed in 2021 at our institution were retrospectively reviewed and a history of prior PCR C+ result recorded. The study protocol was reviewed and approved by the University of California, Irvine Institutional Review Board. Baseline demographics, days from prior C+ result to the surgery date, preoperative methicillin-resistant Staphylococcus aureus (MRSA) nares colonization, and preoperative laboratory markers were obtained to determine baseline characteristics between groups.

All patients received standardized preoperative optimization including weight control and medical co-management when indicated. Within 72 hours prior to surgery, all asymptomatic patients received a COVID test to ensure no active infection or spread of infection through asymptomatic carriers. Patients with C+ results were rescheduled at least four weeks after the last C+ test and retested to ensure negative COVID test 72 hours prior to new surgical date. On the date of surgery, all patients were tested for MRSA Nares in the preoperative area. Patients were then prepped and draped in a standardized fashion including preoperative shaving with electrical clippers as needed and scrub with chlorhexidine gluconate for skin antisepsis. Preoperative prophylaxis included weight-based antibiotic dosing of Ancef, or Vancomycin and Gentamycin for those with penicillin allergies, or for those with a positive MRSA colonization. Of note, the approaches used for the hip procedure were entirely anterior approaches while the approach for knee arthroplasty was the medial parapatellar approach. Postoperatively, patients received two doses of cefazolin 2 grams for 24 hours per standard protocol. Post-surgical venous thromboembolism (VTE) chemoprophylaxis consisted of aspirin 81 mg twice daily for six weeks with the addition of Sequential Compression Devices (SCDs) or compression stockings for patients without any prior history of a DVT. Patients with a history of atrial fibrillation were resumed on their home oral anticoagulant (Apixaban or Rivaroxaban) on postoperative day 1 without aspirin. Patients who were unable to take home oral anticoagulant or aspirin were given daily Lovenox 40 mg injections for six weeks for VTE chemoprophylaxis.

Postoperative estimated blood loss (EBL), length of stay (LOS), rate of revision surgery, and discharge destination were compared between groups. Perioperative and postoperative rates of VTE, urinary tract infection (UTI), pneumonia, postoperative oxygen supplementation, new cardiac arrhythmia, renal disease, sepsis, and periprosthetic joint infections within six months of surgery were recorded. Patients who required any supplemental oxygenation, including nasal cannula or oxygen mask, and patients who required blood transfusion(s) were recorded. Periprosthetic joint infection (PJI) was determined using the updated 2018 criteria for periprosthetic infections including the presence of a sinus tract or two positive cultures with the same pathogen comprising the major criteria, and elevated C-reactive protein (CRP), D-dimer, erythrocyte sedimentation rate (ESR), synovial WBC, Leukocyte esterase, alpha-defensin, synovial polymorphonuclear leukocyte (PMN), synovial CRP comprising minor criteria [10].

Analysis was performed using the SPSS Statistical Tool. Chi-squared tests were used to determine the relationship between prior COVID+ results with postoperative EBL, LOS, rate of revision surgery, discharge destination, rates of VTE, UTI, pneumonia, postoperative oxygen supplementation, cardiac arrhythmia, renal disease, sepsis, and periprosthetic joint infections within six months of surgery. Bivariate logistic regression analysis controlling for baseline demographics was used to determine the relationship between COVID+ diagnosis and association with postoperative complications. Additionally, days from prior C+ diagnosis to surgical date were compared between groups for effects on increased EBL and LOS. Multivariate linear regression was performed to identify COVID positivity as an independent risk factor for postoperative outcomes.

## Results

Of the 155 elective TJA performed in 2021, 24 patients had a prior C+ diagnosis with a mean of 253 days from positive result to surgery date. Of note, the 155 TJA consisted of 93 (60%) knee and 62 (40%) hip arthroplasty. There were no significant differences in comorbidities, BMI, type of postoperative chemical VTE prophylaxis, and WBC/INR preoperative lab markers between groups (Table 1). Although C+ patients had a significantly higher preoperative Hb level, C+ patients were more likely to be males. There were no statistically significant differences with regard to performing surgeon, type of arthroplasty (hip or knee), ASA classification, and smoking status between the two groups.

**Table 1:** Demographics BMI, body mass index; ASA, aspirin; MRSA, methicillin-resistance staphylococcus aureus; WBC, white blood cell; INR, international normalized ratio; Hb, hemoglobin

Demographic Variable	+COVID-19 (n = 24)	-COVID-19 (n = 131)	p
Age, mean ± SD	66.8 ± 9.5	68.0 ± 10.6	0.593
Sex			<0.001
Male, n (%)	18 (75.0)	46 (35.1)	
Female, n (%)	6 (25.0)	85 (64.9)	
BMI, mean ± SD	31.9 ± 5.3	30.4 ± 7.0	0.315
Days From COVID-19+ To Surgery, mean ± SD	253.5 ± 189.3	--	--
Diabetes, n (%)	9 (37.5)	40 (30.5)	0.485
Postoperative Blood Thinner			0.490
ASA, n (%)	21 (87.5)	110 (84.6)	
Lovenox, n (%)	0 (0)	7 (5.4)	
Xarelto/Eliquis, n (%)	3 (12.5)	10 (7.7)	
Multiple, n (%)	0 (0)	3 (2.3)	
Preoperative Nares MRSA+, n (%)	2 (8.3)	2 (1.5)	0.114
Preoperative Laboratory Values			
WBC, mean ± SD	6.4 ± 1.8	7.3 ± 2.4	0.076
INR, mean ± SD	1.0 ± 0.1	1.1 ± 0.3	0.265
Hb, mean ± SD	14.0 ± 1.5	12.8 ± 2.1	0.006

Surgeries on patients with a prior COVID+ had a significantly higher EBL (260 vs 175cc), but postoperative outcomes of VTE, UTI, pneumonia, oxygen supplementation requirement, nares MRSA+, cardiac disease, and infection rates between groups were similar (Tables 2, 3). Of note, no patients within the study required a blood transfusion. Bivariate logistic regression revealed increased days from COVID+ diagnosis (>6 months) to surgery date was associated with a shorter LOS (Table 4). Last, multivariate analysis (Table 5) demonstrated that prior COVID+ diagnosis was associated with greater EBL, and a COVID+ diagnosis cutoff of one year ago was also associated with significantly shorter LOS.

**Table 2:** Postoperative Complications EBL, estimated blood loss; PE, pulmonary embolism; UTI, urinary tract infection.

Postoperative Complication	+COVID-19 (n = 24)	-COVID-19 (n = 131)	p
EBL (mL), mean ± SD	258.3 ± 124.8	175.4 ± 177.9	0.030
PE, n (%)	0 (0)	1 (0.8)	1.000
UTI, n (%)	0 (0)	5 (3.8)	1.000
Postoperative Bleeding or Hematoma, n (%)	0 (0)	5 (3.8)	1.000
Pneumonia, n (%)	0 (0)	1 (0.8)	1.000
Postoperative Nasal Cannula/Oxygen Requirement in Hospital, n (%)	4 (16.7)	7 (5.3)	0.069
Renal Failure, n (%)	0 (0)	3 (2.3)	1.000
Sepsis, n (%)	0 (0)	1 (0.8)	1.000
Cardiac Arrhythmia, n (%)	0 (0)	6 (4.6)	0.591
Periprosthetic Joint Infection, n (%)	0 (0)	4 (3.1)	1.000
Revision Joint Surgery, n (%)	1 (4.2)	3 (2.3)	0.493
Revision for Infection, n (%)	1 (4.2)	3 (2.3)	0.493
Length of Stay, mean ± SD	2.6 ± 1.2	2.5 ± 1.6	0.802
Discharge Destination			0.206
Home, n (%)	18 (75.0)	114 (87.0)	
Rehabilitation, n (%)	6 (25.0)	17 (13.0)	

**Table 3:** Bivariate Logistic Regression for COVID-19 Positivity and Postoperative Complications PE, pulmonary embolism; UTI, urinary tract infection.

Postoperative Complication	OR	95% CI	p
PE	0.000	(0.000, 0.000)	0.998
UTI	0.000	(0.000, 0.000)	0.998
Postoperative Bleeding/Hematoma	0.000	(0.000, 0.000)	0.998
Pneumonia	0.000	(0.000, 0.000)	0.998
Postoperative Nasal Cannula/Oxygen Requirement in Hospital	3.543	(0.950, 13.211)	0.060
Renal Failure	0.000	(0.000, 0.000)	0.998
Sepsis	0.000	(0.000, 0.000)	0.998
Cardiac Arrhythmia	0.000	(0.000, 0.000)	0.998
Periprosthetic Joint Infection	0.000	(0.000, 0.000)	0.998
Revision Joint Surgery	1.855	(0.185, 18.620)	0.599
Revision for Infection	1.855	(0.185, 18.620)	0.599
Discharge to Rehabilitation	2.235	(0.778, 6.421)	0.135

**Table 4:** Bivariate Linear Regression for COVID-19 Positivity and Postoperative Complications Having a history of COVID positivity was associated with significantly greater EBL. As the number of days from COVID-19+ increases, the hospital length of stay decreases. If COVID-19 positivity >6 months or >1 year away from a current hospital stay, LOS significantly decreased. EBL, estimated blood loss; USC B, unstandardized coefficient B.

Postoperative Complication	USC B	95% CI	p
EBL			
COVID-19+	82.913	(7.908, 157.919)	0.030
Days From COVID-19+ To Surgery	-0.170	(-0.451, 0.112)	0.225
COVID-19+ Cutoff 3 Months	-14.737	(-147.610, 118.136)	0.820
COVID-19+ Cutoff 6 Months	-28.571	(-137.427, 80.284)	0.592
COVID-19+ Cutoff 1 Year	-87.500	(-195.381, 20.381)	0.107
Length of Stay			
COVID-19+	0.087	(-0.597, 0.772)	0.802
Days From COVID-19+ To Surgery	-0.003	(-0.006, -0.001)	0.006
COVID-19+ Cutoff 3 Months	-1.032	(-2.241, 0.178)	0.091
COVID-19+ Cutoff 6 Months	-1.229	(-2.144, -0.313)	0.011
COVID-19+ Cutoff 1 Year	-1.437	(-2.352, -0.523)	0.004

**Table 5:** Multivariate Linear Regression for COVID-19 Positivity and Estimated Blood Loss In a multivariate model, COVID-19 positivity was associated with much greater EBL while female sex and ASA use were preoperatively associated with decreased EBL. A COVID-19+ cut-off of 1 year ago was associated with significantly shorter LOS. EBL, estimated blood loss; BMI, body mass index; ASA, aspirin; INR, international normalized ratio; Hg, hemoglobin; USC B, unstandardized coefficient B.

Perioperative Variables	USC B	95% CI	p
EBL			
COVID-19+	78.607	(9.328, 147.887)	0.027
Age	-1.344	(-3.713, 1.025)	0.263
Female Sex	-65.317	(-123.139, 7.495)	0.065
BMI	-2.137	(-6.476, 2.203)	0.331
Diabetes	-49.504	(-106.745, 7.736)	0.089
ASA Perioperative Blood Thinner	-72.002	(-141.647, 2.173)	0.076
INR	0.346	(-108.647, 109.338)	0.995
Hg	-6.301	(-22.296, 9.695)	0.437
Length of Stay			
COVID-19+ Cutoff 1 Year	-1.703	(-3.167, -0.240)	0.026
Age	0.034	(-0.018, 0.086)	0.177
Female Sex	-0.041	(-1.514, 1.433)	0.953
BMI	0.052	(-0.050, 0.155)	0.290
Diabetes	0.171	(-1.439, 1.781)	0.822
ASA Perioperative Blood Thinner	-1.333	(-3.355, 0.689)	0.178
INR	-1.910	(-13.301, 9.480)	0.723
Hg	-0.316	(-0.878, 0.246)	0.246

## Discussion

As the number of TJA performed increases to pre-pandemic rates nationwide, the population of asymptomatic prior C+ patients receiving TJA will increase. Although prior studies have demonstrated higher rates of cardiopulmonary complications, thromboembolic disease, renal injury, and urinary tract infections in postoperative COVID+ patients one month from joint arthroplasty, our study focuses on whether a preoperative resolved asymptomatic COVID+ diagnosis increases the risks for complications and outcomes [6]. Preoperative risk stratification for elective TJA is an important component of perioperative planning and medical optimization in an effort to reduce healthcare costs and decrease preventable complications [11]. As institutions implement COVID testing protocols to prevent the active perioperative spread of COVID in TJA, it is still unknown whether asymptomatic patients have an increased hypercoagulable inflammatory state that may perhaps warrant a prolonged prophylactic course of antibiotics or chemical DVT prophylaxis not routinely prescribed [12]. In this study, we demonstrate preliminary results of a prior COVID+ diagnosis having no increased rates of respiratory, infectious, cardiac, and thromboembolic complications up to six months after elective TJA with the standard postoperative protocol.

While other studies focus on the effects of a new COVID diagnosis during the perioperative period, our study is the first to our knowledge to examine the effects of a prior asymptomatic PCR COVID+ diagnosis > at least 3 months prior to the surgical date. Our average COVID+ diagnosis of ~250 days prior to surgery is relevant for healthcare providers stratifying a rising number of asymptomatic COVID+ elderly patients who have never undergone stresses of surgery post COVID. There are conflicting studies reporting on the prolonged duration of increased DVT, cardiac abnormalities, and PE rates in COVID patients after inoculation, and our study aims to demonstrate asymptomatic patients, >6 months since the last positive PCR test, have no increased risks of UTI, PJI, PE, DVT, and cardiac arrhythmias after joint arthroplasty [13]. Many of our patients had delayed procedures due to positive testing, and they are at increased risk for thromboembolism due to worsened arthritis and reduced mobility during the self-isolation period [14]. Despite theories on increased coagulopathy in prior positive patients, our findings of no increased risks for thromboembolic disease suggest more aggressive prophylactic anticoagulation regimens may not be necessary and otherwise increase the risk for hematoma formation [6,15]. While many of our patients were limited in formal therapy sessions due to pandemic restrictions, our patients were given supplemental standardized home therapy programs to encourage active recovery and mobility.

Although effects of prior COVID diagnosis on respiratory complications have been seen in prior literature, there are no studies correlating PCR COVID diagnosis to either increased MRSA nares colonization or supplemental oxygen requirements in arthroplasty patients. MRSA nares colonization is a known risk factor for periprosthetic joint infections, and prior reports indicate increased MRSA colonization during the COVID pandemic [16]. Our study indicates prior PCR COVID diagnosis had no increased risk for MRSA colonization despite theories on the decreased nasal immune response to respiratory co-pathogens after COVID infection [17]. Our overall low MRSA nares rate may reflect institutionalized trends of mask-wearing, physical distancing, reducing crowds, and hand hygiene used to prevent the spread of respiratory infections. In fact, our COVID patients had no increased leukocytosis or risk for overall UTI, pneumonia, or PJI complications. Low infection rates suggest no overall compromise to the immune function combined with the possible efficacy of current social distancing trends. Our C+ patients not only had no increased rates of MRSA nares colonization and postoperative infections, but they had no increased rates of postoperative oxygen supplementation requirements during their inpatient stay and at physical therapy sessions. Preventing atelectasis is an important postoperative goal to reduce further postoperative hypoxemia that may lead to arrhythmias, myocardial ischemia, and cognitive dysfunction [18]. Prior COVID+ PCR had no effects on post ambulatory breathing oxygenation and no increased rates of nasal cannula use that would indicate reduced respiratory function.

While comorbidities between groups were similar, this study had a higher percentage of males who were COVID+ and subsequently preoperative Hb was higher in the C+ group due to the greater percentages of males [19]. C+ was a significant risk factor for increased EBL intraoperative, which may reflect C+ coagulopathy and loss of antithrombotic mechanisms from imbalances between coagulation and inflammation [12]. While there were no increased postoperative hematomas, INR levels, or postoperative blood transfusions seen in the C+ group, surgeons should strive to obtain meticulous hemostasis and be aware that C+ may increase surgical blood loss. Although discharge destination and LOS were similar between C+ and C-, hospital LOS was inversely related to the number of days from C+ diagnosis to surgical date. COVID-19 positivity >6 months or >1 year away from surgery significantly decreased overall LOS, which may suggest faster recovery and less need for inpatient monitoring. It is possible that our C+ patients with increased EBL combined with inflammatory post-surgical stresses experienced greater physiologic demand postoperatively that required longer inpatient recovery [20]. The findings from this study have important insight for future arthroplasty centers as the surgical community begins to recover from the Covid-19 pandemic, which has caused widespread and numerous delays in surgical care.

There are several limitations to this study. Despite our preliminary findings, suggesting that elective joint replacement surgery is safe in patients with a history of COVID-19, the study is not well powered to detect differences in in-hospital complications, especially for rarer complications such as pulmonary emboli. Additionally, more research is needed in larger samples to confirm the robustness of this finding, as well as to investigate longer-term outcomes. Our cohort of C+ patients may not represent the true spectrum of the disease of all prior C+ patients as our population undergoing elective joint replacement were medically cleared and self-selected to undergo TJA. It is possible that our C+ patients were on the healthier side of the COVID spectrum as sicker patients are more likely to not be medically optimized for elective surgery and be at higher risk of perioperative complications. Since a C+ diagnosis is not randomized and our findings reflect a retrospective review, our results must be viewed as associations and a larger sample size is needed to detect the possible variability in outcomes associated with the various increasing strains of COVID.

## Conclusions

Although a prior COVID+ diagnosis had increased intraoperative blood loss, there were no significant differences in respiratory, infectious, cardiac, and thromboembolic complications up to six months after elective TJA. Increased time from C+ diagnosis to surgical date predicted less EBL and shorter LOS, which may reflect a possible improved recovery in C- compared to C+ patients. This study suggests that asymptomatic C+ patients receiving elective TJA do not require more aggressive prophylactic anticoagulation or antibiotic regimens to prevent VTE or perioperative infections. As institutions around the nation resume pre-COVID rates of arthroplasty surgeries, the effect of prior diagnosis of COVID should be further investigated across a larger sample size to determine the true effect of a prior diagnosis on overall outcomes.
